# The Role of the Ventromedial Prefrontal Cortex in Public Good and Public Bad Games: Evidence From a tDCS Study

**DOI:** 10.3389/fnbeh.2021.666002

**Published:** 2021-08-19

**Authors:** Yuyou Chen, Xinbo Lu, Yuzhen Li, Lulu Zeng, Ping Yu, Jun Luo, Hang Ye, Wanjun Zheng

**Affiliations:** ^1^Center for Economic Behavior and Decision-Making, Zhejiang University of Finance and Economics, Hangzhou, China; ^2^School of Economics, Zhejiang University of Finance and Economics, Hangzhou, China; ^3^School of Economics, Jiaxing University, Jiaxing, China

**Keywords:** cooperation behavior, cooperation belief, ventromedial prefrontal cortex, transcranial direct current stimulation, gains and losses

## Abstract

Although humans constitute an exceptionally cooperative species that is able to collaborate on large scales for common benefits, cooperation remains a longstanding puzzle in biological and social science. Moreover, cooperation is not always related to resource allocation and gains but is often related to losses. Revealing the neurological mechanisms and brain regions related to cooperation is important for reinforcing cooperation-related gains and losses. Recent neuroscience studies have found that the decision-making process of cooperation is involved in the function of the ventromedial prefrontal cortex (VMPFC). In the present study, we aimed to investigate the causal role of the VMPFC in cooperative behavior concerning gains and losses through the application of transcranial direct current stimulation (tDCS). We integrated cooperation-related gains and losses into a unified paradigm. Based on the paradigm, we researched cooperation behaviors regarding gains in standard public good games and introduced public bad games to investigate cooperative behavior regarding losses. Our study revealed that the VMPFC plays different roles concerning gains and losses in situations requiring cooperation. Anodal stimulation over the VMPFC decreased cooperative behavior in public bad games, whereas stimulation over the VMPFC did not change cooperative behavior in public good games. Moreover, participants’ beliefs about others’ cooperation were changed in public bad games but not in public good games. Finally, participants’ cooperative attitudes were not influenced in the public good or public bad games under the three stimulation conditions.

## Introduction

Humans collaborate on large scales and constitute an exceptionally cooperative species that is able to cooperate for common benefits (Gintis, 2003; Boyd and Richerson, 2009; Gächter et al., 2017). However, human cooperation is a complex process and remains a somewhat longstanding evolutionary puzzle that cannot be explained by standard gene-based evolutionary theory (Fehr and Gächter, 2002; Fehr and Fischbacher, 2003; Gintis et al., 2003; Colman, 2006; Boyd and Richerson, 2009; Foley and Gamble, 2009). In general, the major evolutionary mechanisms that have been proposed to explain human cooperation include kinship, reciprocity, reputation, signaling, and punishment (Henrich and Muthukrishna, 2021).

To further observe and explain cooperation behavior, a series of economic experiments were proposed, such as the prisoner’s dilemma, the tragedy of the commons, and public good games. Although prosocial motives are one of the most important reasons for higher levels of cooperation than those that maximize personal benefits (Burton-Chellew and West, 2013), evidence has shown that prosocial preferences are not always pervasive enough to eliminate free-riding in all cultures (Herrmann et al., 2008; Krajbich et al., 2009). Moreover, conditional cooperation and individuals’ beliefs about others’ cooperation were also important in the process of providing and maintaining cooperation (Fischbacher et al., 2001; Kocher et al., 2008; Gächter et al., 2017). Theories from biology, economics, and psychology revealed that a behavioral pattern of cooperating only with those who display cooperative behavior can spread over a society (Nowak and Sigmund, 1998; Bshary and Grutter, 2006; Fudenberg and Maskin, 2009; Suzuki et al., 2011). Cooperation behaviors are also related to reputation. Indirect reciprocity theory proposes that everyone in a group is continually assessed and that cooperation is channeled toward the “valuable” members of the community (Nowak and Sigmund, 1998; Wedekind and Milinski, 2000; Leimar and Hammerstein, 2001). Costly signaling theory suggests that cooperation evolves because it involves an honest signal of the community member’s quality and therefore results in advantageous alliances (Gintis et al., 2001; Higham, 2014; McAndrew, 2018).

Clearly, most of the studies above adopted gain paradigms, such as public good games, trust, and reciprocity games, prisoners’ dilemmas, and stag hunt games, to investigate cooperation behavior (Axelrod, 1980; Berg et al., 1995; Anderson et al., 1998; Fehr and Gächter, 2000; Rankin et al., 2000). However, cooperation is not always related to resource allocation and gains but is often related to reducing losses. Environmental pollution, carbon dioxide emissions, and refuse disposal are another kind of cooperation problems (Hardin, 2009; Gächter et al., 2017).

In accordance with behavioral studies and theoretical explanations, recent neuroimaging and electroencephalogram (EEG) studies have revealed that the decision-making process of cooperation is involved in the function of many brain areas (McCabe et al., 2001; Frith and Singer, 2008; Rilling et al., 2008; Baumgartner et al., 2012; Chung et al., 2015). Neuroscience research found that cooperation was associated with consistent activation in brain regions linked with the reward system: the caudate nucleus, ventromedial prefrontal cortex (VMPFC), anterior cingulate cortex, and medial prefrontal cortex (MPFC; Rilling et al., 2002; Baumgartner et al., 2012; Bault et al., 2015; Van Hoorn et al., 2016; Park et al., 2019). Electroencephalogram (EEG) studies proposed that neural oscillations in centroparietal and temporal regions had the highest power in predicting individuals’ decisions in public good games (Chung et al., 2015). Psychopathy studies have shown that cooperative behavior in subjects with higher psychopathy was accompanied by activation within the orbitofrontal cortex and defective behavior was accompanied by weaker activation within the dorsolateral prefrontal and rostral anterior cingulate cortex (Rilling et al., 2007). Furthermore, conditional cooperation was also studied. In a study of prisoner dilemma games, it was revealed that the right dorsolateral prefrontal cortex manifested greater activation when subjects confronted noncooperative opponents (Suzuki et al., 2011).

In addition to revealing neural activity associated with cooperation, neuroimaging studies have investigated related brain regions concerned with subjects’ beliefs about others’ cooperation. Generally, four cortical regions are dedicated to components of the process of perceiving information about others: the MPFC, the right and left temporoparietal junctions (RTPJ and LTPJ, respectively), and the posterior cingulate (PC; Frith and Frith, 2003; Gallagher and Frith, 2003; Rilling et al., 2004; Saxe and Wexler, 2005; Heatherton et al., 2006; Park et al., 2019). In particular, Yoshida et al., 2010 revealed that the MPFC is involved in encoding uncertainty inferences about others’ decisions during cooperative games. On the basis of fMRI data acquired regarding public good games, Park et al. (2019) showed that the TPJ and the anterior cingulate cortex updated beliefs about others’ decisions during an interaction.

Because cooperation also involves losses, neuroimaging studies have also investigated related brain regions concerned with subject loss. Recent studies revealed that the gain and loss network included brain regions associated with anticipation and receipt of rewards, including the dorsal and ventral striatum, VMPFC, ventrolateral PF, anterior cingulate cortex, and dopaminergic midbrain regions (Tom et al., 2007). Moreover, the VMPFC is believed to play an important role in mediating value-based decision-making (Pujara et al., 2015).

Although many brain regions, such as the caudate nucleus, anterior cingulate cortex, TPJ, and PC, are important for cooperative behavior, the role of the VMPFC is critical in the process of decision making and defining beliefs about others in cooperation games. However, neuroimaging and electroencephalogram (EEG) studies allow us to identify the association between neural activity and cooperative behaviors, and the direct causal relationship remains unknown and requires further confirmation. Fortunately, “virtual lesions” are created by using stimulation technologies such as transcranial direct current stimulation (tDCS), repetitive transcranial magnetic stimulation (rTMS), and continuous theta-burst stimulation (cTBS) to provide a convenient way to identify causal relationships between cooperative behavior and the target brain region. Regarding tDCS, Gerfo et al. (2019) established that anodal tDCS over the VMPFC increased punishment behavior in situations requiring cooperation. Zheng et al. (2016) demonstrated that activating the VMPFC could promote subjects’ altruism preference in trust games. Building on the previous neuroimaging and EEG data, we aimed to investigate the causal role of the VMPFC in cooperative behavior and beliefs about others’ cooperation.

In this study, we integrated gain and loss into a unified paradigm. Specifically, we discussed cooperation behaviors in gains in standard public good games and introduced public bad games to investigate cooperation behaviors in losses. Based on this paradigm, we investigated the causal role of the VMPFC in cooperative behavior concerning gains and losses through the application of tDCS. Finally, whether stimulation of the VMPFC can change individuals’ attitudes and beliefs concerning cooperation under gain and loss conditions are necessary to be examined.

## Materials and Methods

### Subjects

We recruited a total of 180 healthy students (102 females; mean age of 20.3 years, ranging from 17 to 25 years) from Zhejiang University of Finance and Economics. All participants met the following conditions: right-handed; unfamiliar with tDCS; and no history of clinical impairments, psychiatric illness, or neurological disorders. The participants were randomly assigned to play either public good games (*n* = 84, 49 females) or public bad games (*n* = 96, 53 females). In the public good games group and public bad games group, the participants were randomly assigned to receive sham stimulation (*n* = 28, 18 females; *n* = 32, 19 females), anodal tDCS (*n* = 28, 15 females; *n* = 32, 18 females), or cathodal tDCS (*n* = 28, 16 females; *n* = 32, 16 females). Participants received a fixed show-up fee of 10 CNY (approximately 1.54 US dollars) in addition to the money they gained in the public good game or public bad game. The entire experiment lasted approximately 55 min, and on average, participants received a payment of approximately 58.7 CNY (approximately 9.06 US dollars) from the games, ranging from 41 to 72 CNY according to their performance. Participants gave written informed consent before entering the study, which was approved by the Zhejiang University of Finance and Economics Ethics Committee. No participants reported any adverse side effects involving pain on the scalp or headaches.

### Transcranial Direct Current Stimulation (tDCS)

tDCS was achieved by applying a weak direct current to the scalp *via* two saline-soaked surface sponge electrodes. The current was constant and delivered by a battery-driven stimulator (multichannel, noninvasive wireless tDCS neurostimulator, Starlab, Barcelona, Spain), which was controlled by a Bluetooth system. Generally, cathodal stimulation restrains cortical excitability, whereas anodal stimulation enhances it (Nitsche and Paulus, 2000).

**Figure 1:**
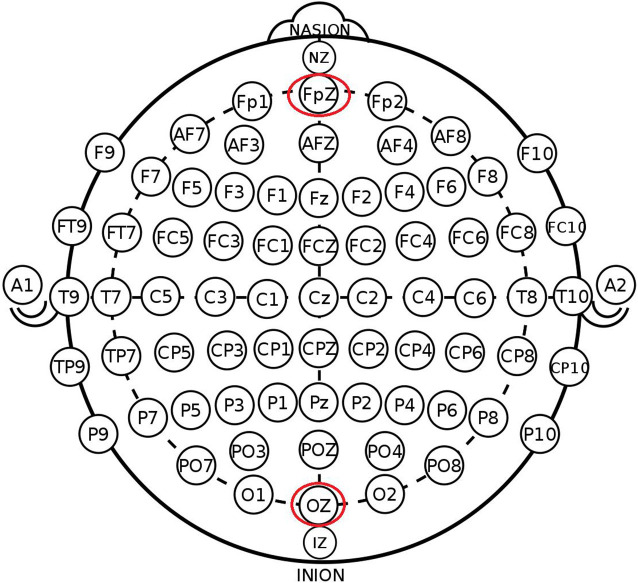
Schematic and locations of electrode positions.

**Figure 2:**
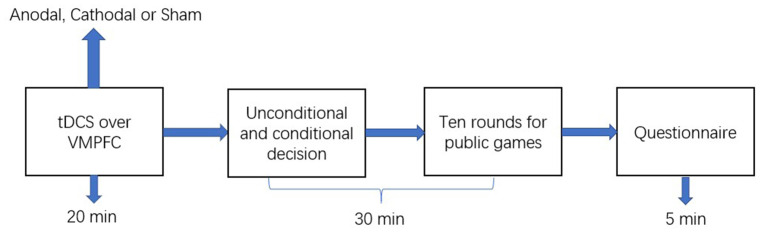
Schematic represent of the experiment design. After 20 min of stimulation, the participant was asked to complete public good or public bad games.

The participants were randomly assigned to one of the three stimulation conditions: (1) anodal stimulation over the VMPFC (Figure 1); (2) cathodal stimulation over the VMPFC; and (3) sham stimulation. A constant current of 1.5 mA to fPZ was applied for 20 min. Electrode positions were established through the EEG 10–20 International System. Specifically, the center of the anode or cathode was positioned on the FpZ site. The anode (3 cm * 3 cm) was placed on the FpZ site, and the cathode (5 cm * 7 cm) was placed over the OZ site under anodal stimulation conditions to increase the focality of the stimulation (Nitsche et al., 2008). In contrast, the cathode (3 cm * 3 cm) was placed on the FpZ site, and the anode (5 cm * 7 cm) was placed over the OZ site under cathodal stimulation conditions.

The electrical field was strong under both anodal and cathodal electrodes. Following the standard tDCS protocol, stimulation commenced after a 30 s ramp-up period, and the current was ramped down over the last 30 s. For sham stimulation, the current lasted only 30 s. This has proven to be reliable because the brief duration of stimulation hardly modulates cortical excitability, but the participants may feel the initial itching and believe they were receiving stimulation (Gandiga et al., 2006).

### Experimental Task and Procedure

#### The Public Good Experiment and the Public Bad Experiment

The public good experiment was composed of two stages, and the decision situation was a standard linear public good choice. Each of the four individuals had an endowment of 20 tokens. Each individual could either keep these tokens for herself or invest them in a project. The incentives for each individual were explained by the following equation:

(1) $$\pi_{i}=20-g_{i}+0.4\sum_{j=1}^{4}g_{j}$$

Specifically, *g*_i_ denotes the contribution of individual i to the project, and the marginal payoff of a contribution to the public good is 0.4 tokens, which is consistent with previous studies (Andreoni, 1995a; Anderson et al., 1998). In the first stage, individuals were asked to make two types of contribution decisions. The first type of decision was called an unconditional contribution, and the second type of decision was called a contribution table, which was consistent with previous studies (Fischbacher et al., 2001; Gächter et al., 2017). In the unconditional decision, individuals were asked how many of their 20 tokens they wanted to contribute to the common pool. In the conditional decision, individuals had to fill out a contribution table in which they were to indicate their contribution for each of 21 possible average contribution levels compared to the contributions of the other three group members. For the purpose of incentive, for three random members in each group, the unconditional contribution was designated as the relevant contribution. For the other member, the contribution table became her relevant decision. In the second stage, participants were randomly matched in groups of four and played for 10 consecutive rounds under payment rules that followed the above equation. At the end of every round, participants were asked to guess the average contributions of the other three group members. To achieve incentive compatibility, participants were paid one token if their guesses were correct. In addition, participants were not told how many rounds the experiment would last.

The public bad experiment was also composed of two stages. Each of the four individuals had an endowment of 52 tokens and faced a loss of 20 tokens. Each individual could either bear the loss by herself or put them into a project. The incentives for each individual were explained by the following equation:

(2) $$\pi_{i}=52-(20-b_{i})-0.4\sum_{j=1}^{4}b_{j}$$

Specifically, *b*_i_ denotes the loss individual i put into the project. Individuals’ personal loss was 20 − *b*_i_ after they put a loss of *b*_i_ into the project, and the marginal payoff of a loss to the public bad was 0.4 tokens. In the first stage, the individuals were asked to make two types of loss decisions (unconditional and conditional decisions), which was similar to the procedure in the public good games. In the second stage, participants played for 10 consecutive rounds under the payment rules of equation (2). Similar to public good games, participants were asked to guess the average loss contributions of the other three group members in all rounds. The design of bad public experiments was inspired by previous studies about the framing of public good games (Andreoni, 1995b; Sonnemans et al., 1998; Dufwenberg et al., 2011; Gächter et al., 2017), which mainly paid attention to the aspect of giving and taking behaviors and focused on providing and maintaining common resources. The present study paid attention to gains and losses and focused on the public good and the tragedy of the commons, which was consistent with previous studies such as that by De Dreu and McCusker (1997).

#### Experimental Procedure

The experimental software *z*-Tree was used to present the public good and public bad games as well as to calculate participants’ final payoff (Fischbacher, 2007). The whole experiment was performed in three phases (Figure 2). In the first phase, the participants received stimulations for 20 min (anodal, cathodal, or sham stimulation). In the second phase, participants completed the public good or public bad games. Furthermore, when participants took part in the second stage of the public good or public bad games, they did not know the result of the first stage of the public good or public bad games. Additionally, participants had to pass a control question test before taking part in the games. The control question test was conducted to ensure that every participant understood the games before participating in the games. In the third phase, after the participants completed the public good or public bad games, they were asked to complete a questionnaire before they received their final payment. The questionnaire contained questions about their personal information, such as gender, age, and consumption expenditure.

## Data Analysis

Participants’ conditional decisions in the strategy-method experiment are critical variables for investigating their cooperative attitudes. Consistent with previous studies (Fischbacher et al., 2001; Gächter et al., 2017), we classified participants’ cooperative attitudes into three main types. The first type was free riders; this type of participant contributed 0 in public good games or put a loss of 20 tokens in public bad games regardless of how much the others contributed. The second type was conditional cooperators; for these participants, the Spearman’s correlation coefficient between their contribution schedule and the other’s average contribution was significantly positive in public good or bad games. The third type was designated as “other” when neither the first type nor the second type applied. To be specific, the type of “others” mainly refers to the “hump-shaped” contributions (Fischbacher et al., 2001). In this condition, participants are close to perfect conditional cooperation for contribution levels of up to 10 tokens of the other group members. However, beyond this level they steadily reduce their contributions. Therefore, the type of others does not apply to “free riders” or “conditional co-operators”. Cooperation attitudes revealed one’s willingness to cooperate as a function of other members’ cooperation.

After obtaining participants’ cooperative attitudes, we concentrated on examining the public good and bad experiment outcomes. Then, we investigated participants’ beliefs about other group members’ cooperation by using the estimated contributions in public good and bad games. Cooperative behaviors and beliefs about cooperation were not normally distributed, as assessed by the Shapiro-Wilk test. To test the causal relationship between the activity of the VMPFC and participants’ cooperation behaviors, we conducted the Kruskal-Wallis test to determine whether there were differences in the amount between the three kinds of stimulations. When a significant difference was found, *post hoc* analyses (H test) were performed to identify specific differences. Finally, the Spearman test was applied to examine the correlation between observed cooperative behaviors and estimated cooperative behaviors.

All data were statistically evaluated using Stata software. The significance level was set at 0.05 for all analyses. Means and standard errors of cooperation behaviors and estimated cooperation behaviors in public good games and public bad games are shown in Tables s 1–4.

**Table 1:** Means and SE of the data for contributions in public good games under three conditions.

Stimulation	Round1	Round2	Round3	Round4	Round5	Round6	Round7	Round8	Round9	Round10
Anodal	6.18	4.18	3.04	2.18	2.18	2.07	2.68	2.14	1.82	1.61
	(1.09)	(0.89)	(0.80)	(0.56)	(0.66)	(0.58)	(0.68)	(0.71)	(0.71)	(0.65)
Sham	4.93	4.79	5.21	4.29	3.54	3.82	3.54	3.29	3.11	3.14
	(0.77)	(0.89)	(1.18)	(1.14)	(1.07)	(1.17)	(1.09)	(1.10)	(1.05)	(1.04)
Cathodal	5.18	5.36	3.54	2.18	2.50	3.18	2.54	2.50	2.54	1.96
	(0.98)	(1.05)	(0.93)	(0.58)	(0.62)	(0.86)	(0.64)	(0.63)	(0.74)	(0.66)

**Table 2:** Means and SE of the data for estimated contributions in public good games under three conditions.

Stimulation	Round1	Round2	Round3	Round4	Round5	Round6	Round7	Round8	Round9	Round10
Anodal	6.46	5.18	4.32	3.21	2.64	2.00	2.54	2.36	1.86	2.00
	(0.93)	(0.60)	(0.69)	(0.47)	(0.51)	(0.45)	(0.52)	(0.58)	(0.61)	(0.63)
Sham	6.18	6.07	5.00	4.71	3.25	3.07	2.82	3.11	3.04	3.11
	(0.80)	(0.81)	(0.97)	(1.01)	(0.91)	(0.82)	(0.77)	(0.85)	(0.87)	(0.89)
Cathodal	5.64	5.79	4.29	3.93	3.36	3.46	3.11	3.21	2.71	2.36
	(0.78)	(0.86)	(0.78)	(0.75)	(0.67)	(0.63)	(0.56)	(0.67)	(0.67)	(0.71)

**Table 3:** Means and SE of the data for loss contributions in public bad games under three conditions.

Stimulation	Round1	Round2	Round3	Round4	Round5	Round6	Round7	Round8	Round9	Round10
Anodal	16.47	17.25	17.29	17.57	18.71	18.64	19.35	19.04	20	19.0
	(1.12)	(0.84)	(1.08)	(1.07)	(0.57)	(0.82)	(0.45)	(0.73)	(0)	(0.73)
Sham	12.97	14.90	15.8	16.43	16.46	16.2	16.0	17.7	17.5	17.9
	(1.20)	(1.21)	(1.15)	(1.19)	(1.23)	(1.18)	(1.21)	(0.98)	(0.96)	(0.96)
Cathodal	13.97	17.09	17.60	18.25	18.57	18.32	17.86	18.14	18.57	17.24
	(1.28)	(0.90)	(0.68)	(0.80)	(0.57)	(0.78)	(1.00)	(0.87)	(0.75)	(1.10)

**Table 4:** Means and SE of the data for estimated loss contributions in public bad games under three conditions.

Stimulation	Round1	Round2	Round3	Round4	Round5	Round6	Round7	Round8	Round9	Round10
Anodal	14.78	14.93	16.78	17.36	16.75	17.25	18.04	17.82	18.78	18
	0.99	0.92	0.95	0.69	0.97	0.95	0.81	0.86	0.59	0.67
Sham	9.78	14.09	14.83	16.03	15.43	17.33	15.93	16.83	17.47	17.67
	1.15	0.80	0.75	0.80	1.04	0.76	1.07	0.89	0.63	0.80
Cathodal	11.09	13.93	16.32	16	16.46	16.43	16.21	16.96	15.75	15.89
	(1.30)	(1.14)	(1.06)	(1.29)	(1.11)	(1.12)	(1.25)	(1.11)	(1.32)	(1.24)

## Results

### Public Good and Public Bad Experiment Outcomes in the Sham Group

**Figure 3:**
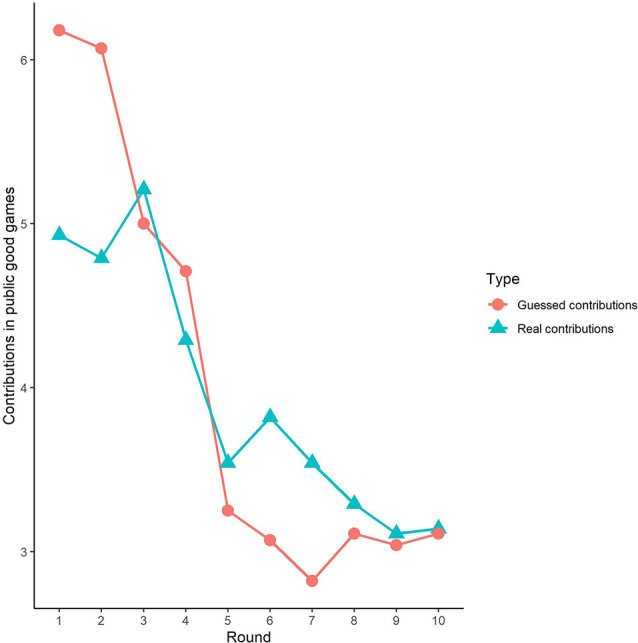
Real contributions and guessed contributions by round in public good games.

**Figure 4:**
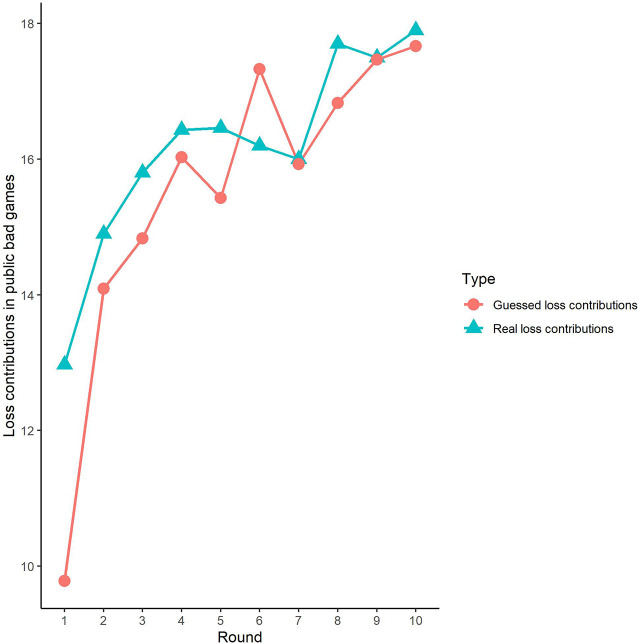
Real contributions and guessed contributions by round in public bad games.

First, we examined the public good experiment outcomes in the sham group over 10 rounds (Figure 3). As shown in Figure 4, cooperation in the public good experiment had a tendency to decline, with a contribution of 4.93 in the first round, decaying to 3.14 by round 10, which is consistent with previous studies (Fischbacher et al., 2001; Gächter et al., 2017). Similarly, the estimated reciprocity had a tendency to decrease, with an estimated contribution of 6.18 in round 1, decaying to 3.11 by round 10. Moreover, the estimated contribution was higher than the real contribution in round 1 and lower in round 10. This result indicated that the estimated contribution decreased faster than the real contribution. Finally, we investigated cooperation attitudes. The results showed that 21.4% of subjects can be classified as free riders, 53.6% as conditional cooperators, and 25% as others.

Second, we further examined the public bad experiment outcomes in the sham group over 10 rounds (Figure 4). As shown in Figure 4, the loss contribution in the public bad condition had a tendency to increase, with a loss contribution of 12.97 in round 1, increasing to 17.90 by round 10, which was contrary to the contribution in the public good condition. Similarly, the estimated loss contribution had a tendency to increase, with an estimated loss contribution of 9.78 in round 1, increasing to 17.67 by round 10. Moreover, the estimated loss contribution was lower than the real loss contribution over all 10 rounds. We further investigated cooperation attitudes. The results showed that 40.6% of the subjects can be classified as free riders, 34.4% as conditional cooperators, 25% as others.

Third, we investigated the correlation between observed cooperative behaviors and estimated cooperative behaviors. In public good games, Spearman’s test results revealed that actual contributions were highly significantly positively correlated with predicted contributions in round 1 (Spearman’s rho = 0.61, *p* < 0.001). Similarly, the actual loss contributions in public bad games were highly significantly positively correlated with predicted loss contributions (Spearman’s rho = 0.51, *p* < 0.001). We also investigated the correlations from round 2 to round 9, and we found that cooperative behaviors and estimated cooperative behaviors were significantly positively correlated in both the public good and bad conditions (*p* < 0.01).

### Contribution in the Public Good Games: the Stimulation Effect

**Figure 5:**
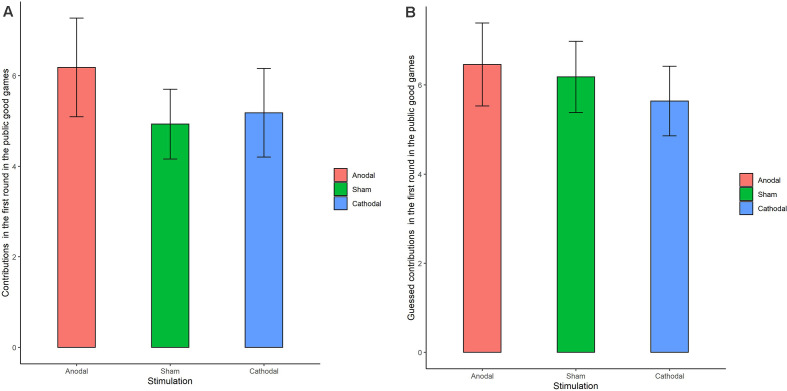
Real contributions **(A)** and guessed contributions **(B)** in the first round for public good games in three stimulation conditions. Error bar represents standard error.

The Shapiro-Wilk test showed that contributions to the public good were not normally distributed (*p* < 0.001). To test the stimulation effect, we adopted the Kruskal-Wallis test to determine whether there was a difference in the amount contributed to the public good. The Kruskal-Wallis test revealed that there was no significant difference in the contribution amount in round 1 among the three stimulation conditions (Figure 5A; $\chi_{d.f.2}^{2}$ = 0.374, *p* = 0.829). There was also no significant difference in the contribution amounts from round 2 to round 10 (*p* > 0.1 in all the rounds). We further examined the estimated contribution amounts among the three stimulation conditions. The Kruskal-Wallis test revealed that there was no significant difference in the estimated contribution amount over the 10 rounds among the three stimulation conditions (Figure 5B; *p* = 0.761 in the first round and *p* > 0.1 in all the other rounds). In addition, no significant difference was found in individuals’ cooperative attitudes among the three stimulation conditions ($\chi_{d.f.2}^{2}$ = 2.76 *p* = 0.599).

### Loss Contributions in the Public Bad Games: the Stimulation Effect

**Figure 6:**
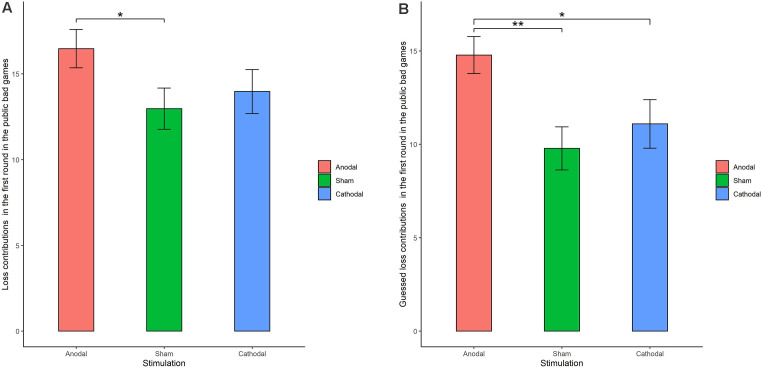
Real contributions **(A)** and guessed contributions **(B)** in the first round for public bad games in three stimulation conditions. Error bar represents standard error. *p < 0.05, **p < 0.01.

The Shapiro-Wilk test showed that loss contributions in the public bad were not normally distributed (*p* < 0.001). To test the stimulation effect, we employed the Kruskal-Wallis test to determine whether there was a difference in the amount contributed to the public bad among the three stimulation conditions. The results revealed that there was a significant difference in the amount contributed to the public bad in round 1 (Figure 6A; $\chi_{d.f.2}^{2}$ = 0.046, *p* = 0.046). Post hoc analysis (H test) showed that loss contributions significantly increased after receiving anodal stimulation compared with sham stimulation in round 1 (FDR-adjusted, *p* = 0.012). Although loss contributions increased after receiving anodal stimulation compared with the contributions observed after cathodal stimulation, the difference was not significant (FDR-adjusted, *p* = 0.075). However, the Kruskal-Wallis test revealed that there was no significant difference in loss contributions among the three conditions from round 2 to round 10 (*p* > 0.1). This finding indicated that anodal stimulation in the VMPFC made participants less cooperative at the beginning of public bad games.

We further examined whether the estimated loss contributions were affected by stimulation. The Kruskal-Wallis test revealed that there was a significant difference in the estimated loss contribution in round 1 among the three stimulation conditions (Figure 6B; $\chi_{d.f.2}^{2}$ = 9.66, *p* < 0.01). Post hoc analysis (H test) showed that the estimated loss contribution significantly increased after receiving anodal stimulation compared with sham stimulation in round 1 (FDR-adjusted, *p* < 0.01). Post hoc analysis (H test) also showed that the estimated loss contribution significantly increased after receiving anodal stimulation compared with the loss contribution observed after cathodal stimulation in round 1 (FDR-adjusted, *p* = 0.019). However, the estimated loss contribution after receiving cathodal stimulation was higher than that after receiving sham stimulation in round 1, but the difference was not significant (FDR-adjusted *p* = 0.190).

The results indicated that anodal stimulation in the VMPFC changed participants’ beliefs about other participants’ behavior in round 1. However, the Kruskal-Wallis test revealed that there was no significant difference in the estimated loss contributions from round 2 to round 9 among the three stimulation conditions (*p* > 0.01). In addition, no significant difference was found in individuals’ cooperative attitudes among the three stimulation conditions ($\chi_{d.f.4}^{2}$ = 1.84, *p* = 0.765).

## Discussion

Although humans constitute an exceptionally cooperative species that is able to collaborate on large–scales for common benefits, the cooperative behavior of humans remains a longstanding puzzle to some extent. As cooperative behavior entails a complex decision-making process, a large body of previous studies from different fields has discussed the issues of cooperation. Many neuroscience studies have shown that many brain regions, such as the caudate nucleus, VMPFC, anterior cingulate cortex, and TPJs (McCabe et al., 2001; Frith and Singer, 2008; Rilling et al., 2008; Baumgartner et al., 2012; Chung et al., 2015), have been implicated in cooperative behavior. Evidence also shows that the VMPFC is a crucial region concerned with cooperative attitudes and beliefs about others’ cooperation. Moreover, cooperation is not only connected with contributions to common resources but also related to losses. The current study investigated the causal role of the VMPFC in cooperative behavior in gains and losses by integrating public good and public bad games into a unified paradigm.

At the behavior level, the results showed that contributions in public good games had a tendency to decay over all 10 rounds. In contrast, loss contributions in public bad games had a tendency to increase over all 10 rounds. In the first round of public good games, the estimated contributions of others are lower than real contributions. This changed participants’ beliefs in cooperation. Then the estimated contributions of others and real contributions decreased in the later rounds of public good games. In contrast, the estimated loss contributions of others are higher than real contributions. This also changed participants’ beliefs in others in cooperation. Then the estimated loss contributions of others and real contributions increased in the later rounds of public bad games.These findings are in line with previous literature, which showed that cooperation behaviors decline to a lower level over all rounds (Fischbacher et al., 2001; Gächter et al., 2017).

Second, the estimated contributions of others declined in public good games and increased in public bad games. Moreover, the estimated contributions decreased faster than the actual average contributions in public good games, whereas the estimated contributions were always lower than the actual average contributions in public bad games. A possible reason is that the subjects’ attitudes toward losses and gains are different (Tversky and Kahneman, 1991; Tom et al., 2007). In general, losses loom larger than gains. The different attitudes towards losses and gains may finally lead to different biases toward the intentions of others in the public good and bad games. We also found that actual contributions were highly significantly positively correlated with estimated contributions in the public good and bad games. This finding indicated that the decreased cooperation levels were related to the decay in the beliefs about others’ cooperation, which is consistent with previous studies (Fischbacher et al., 2001; Gächter et al., 2017).

Regarding the effect of tDCS applied to the VMPFC on cooperative behaviors, our data revealed that the VMPFC differentially modulates cooperation behavior in the public good and public bad games. Indeed, though anodal stimulation over the VMPFC decreased cooperative behaviors at the beginning of public bad games, anodal or cathodal stimulation did not significantly change cooperative behaviors over public good games. In general, the effect of anodal stimulation is more obvious than cathodal stimulation in both the public good and bad games. Recent neuroimaging studies have demonstrated that the VMPFC is a critical part of the reward system (De Quervain et al., 2004; Hu et al., 2015; Zinchenko and Arsalidou, 2018; Gerfo et al., 2019). Moreover, the VMPFC encodes immediate expected rewards as individual utility, whereas the lateral frontal cortex encodes group utility (Park et al., 2019). Neuroimaging and lesion studies have revealed that the VMPFC plays a key role in mediating value-based decision making (Tom et al., 2007; Pujara et al., 2015). Tom et al. (2007) proposed that the VMPFC exhibited a pattern of neural loss aversion in gain and loss conjunction analysis. Our results indicated that the VMPFC plays different roles in gains and losses in cooperation, which is in line with previous studies.

To further interpret the mechanism of cooperative behavior in the public good and public bad games, subjects’ beliefs about others’ cooperation and about cooperative attitudes were evaluated. Spearman’s test indicated that cooperative behavior was positively correlated with subjects’ beliefs about others’ cooperation. This is consistent with previous literature (Fischbacher et al., 2001; Kocher et al., 2008; Gächter et al., 2017). Based on the correlation of cooperative behaviors and beliefs about cooperation, we further investigated the effect of stimulation on beliefs about cooperation. Our results revealed that anodal stimulation decreased subjects’ beliefs about others’ cooperation compared with the beliefs observed after cathodal stimulation or sham stimulation at the beginning of public bad games. However, the data revealed no significant differences in subjects’ beliefs about cooperation in public good games under the three stimulation conditions. This finding seems to indicate that the VMPFC plays different roles in beliefs about others’ cooperative behaviors concerning gains and losses. Regarding cooperative attitudes, our data revealed that subjects’ cooperative attitudes were not changed under the three stimulation conditions. Thus, VMPFC stimulation changed cooperative behavior and beliefs about cooperation but did not affect subjects’ cooperative attitudes regarding conditional cooperation in public bad games. Nevertheless, the VMPFC neither changed cooperative behavior and beliefs about cooperation nor changed cooperative attitudes regarding conditional cooperation in public good games. Previous studies have revealed that the VMPFC is associated with altruism. According to clinical lesion studies, altruistic behaviors were weakened in patients with damage to the VMPFC (Krajbich et al., 2009; Moretto et al., 2013). However, as cooperation is related to cooperative attitudes and beliefs about others’ cooperation, cooperative behavior is a more complex decision-making process than altruism, as shown in previous studies (Fischbacher et al., 2001; Kocher et al., 2008; Gächter et al., 2017) and in our studies. Furthermore, previous studies found that the MPFC is dedicated to the process of perceiving information about others (McCabe et al., 2001; Yoshida et al., 2010). Heatherton et al. (2006) proposed that medial prefrontal activity differentiates the self from close others. Yoshida et al. (2010) showed that the rostral MPFC has a specific role in encoding the uncertainty of inference about others’ strategies. Our results support the previous fMRI data and seem to indicate that the VMPFC plays a critical role in inferencing information concerning others’ cooperation strategies in loss conditions.

Although our findings revealed that altering excitability in the VMPFC changed participants’ cooperative behavior and beliefs about cooperation in public bad games, the current study has some relevant limitations. First, the neural circuitry underlying the decision-making process of behavior, beliefs, and attitudes regarding cooperation cannot be demonstrated by a single experiment. Moreover, the prefrontal cortex (PFC) is widely viewed as a source of this inhibitory control. A prevalent view is that certain PFC regions are specialized for inhibitory control. For example, the right inferior frontal gyrus (rIFG) is a specialized response inhibition area, the ventromedial prefrontal cortex (vmPFC) supports coping with controllable stressors, and the right middle frontal gyrus (rMFG) seems to exert inhibitory control over memory-related areas (Munakata et al., 2011). The decision process in public games may be that anodal stimulation simply removes the inhibitory system and leads to an increase decreasing cooperation in public bad games. Future research may focus on the inhibitory effect of different PFC regions in the public good and bad games. Second, tDCS may have an effect on not only ventromedial regions but also lateral and dorsal regions in the anodal and cathodal stimulations. Cooperation involves executive functions and mentalizing abilities. The orbitofrontal cortex has a fundamental role in making behavioral choices, particularly in incompletely specified or unpredictable situations (Elliott et al., 2000; Decety et al., 2004). In addition, medial prefrontal cortex activation is associated with mentalizing tasks (Decety et al., 2004). Moreover, DLPFC is linked to social tactics and strategic interaction in cooperation games (Emonds et al., 2012). Therefore, future studies may want to focus on examining the role of other prefrontal areas in cooperative behavior. Third, the involvement of other brain areas, such as the TPJ. Therefore, future studies may want to focus on examining other brain regions and the neural circuitry of the VMPFC. Fourth, confounding biases may arise from two electrodes with opposite polarities over the scalp. The effects of the adopted tDCS montage should be compared with a fronto-extracephalic montage in future studies. For example, a control group using an “extracephalic electrode reference” (one placed over the scalp and the other over the right deltoid muscle) could be included to reduce the confounding effect of a fronto-occipital montage. Finally, a within-subject design may have had the advantage of comparing cooperative behaviors in the public good and public bad games. Furthermore, future studies should adopt neuroimaging measures and rTMS to study the neural changes associated with cooperative behavior in the public good and public bad games.

## Data Availability Statement

The raw data supporting the conclusions of this article will be made available by the authors, without undue reservation.

## Ethics Statement

The studies involving human participants were reviewed and approved by Zhejiang University of Finance and Economics Ethics Committee. The patients/participants provided their written informed consent to participate in this study.

## Author Contributions

YC and WZ: conceptualization. YC, WZ, JL, XL, and HY: methodology. WZ, YL, LZ, and YC: validation. YC, WZ, and PY: writing—review and editing. All authors have read and agreed to the published version of the manuscript.

## Conflict of Interest

The authors declare that the research was conducted in the absence of any commercial or financial relationships that could be construed as a potential conflict of interest.

## Publisher’s Note

All claims expressed in this article are solely those of the authors and do not necessarily represent those of their affiliated organizations, or those of the publisher, the editors and the reviewers. Any product that may be evaluated in this article, or claim that may be made by its manufacturer, is not guaranteed or endorsed by the publisher.
